# Supplementary material for: Phosphorylation of Syntaxin 4 by the Insulin Receptor Drives Exocytic SNARE Complex Formation to Deliver GLUT4 to the Cell Surface
Source: Biomolecules. 2023 Dec 2;13(12):1738. doi: 10.3390/biom13121738 (PMC10741561; doi:10.3390/biom13121738)

Figure S1. Original Western Blotting Figures of Figure 1A,B

A

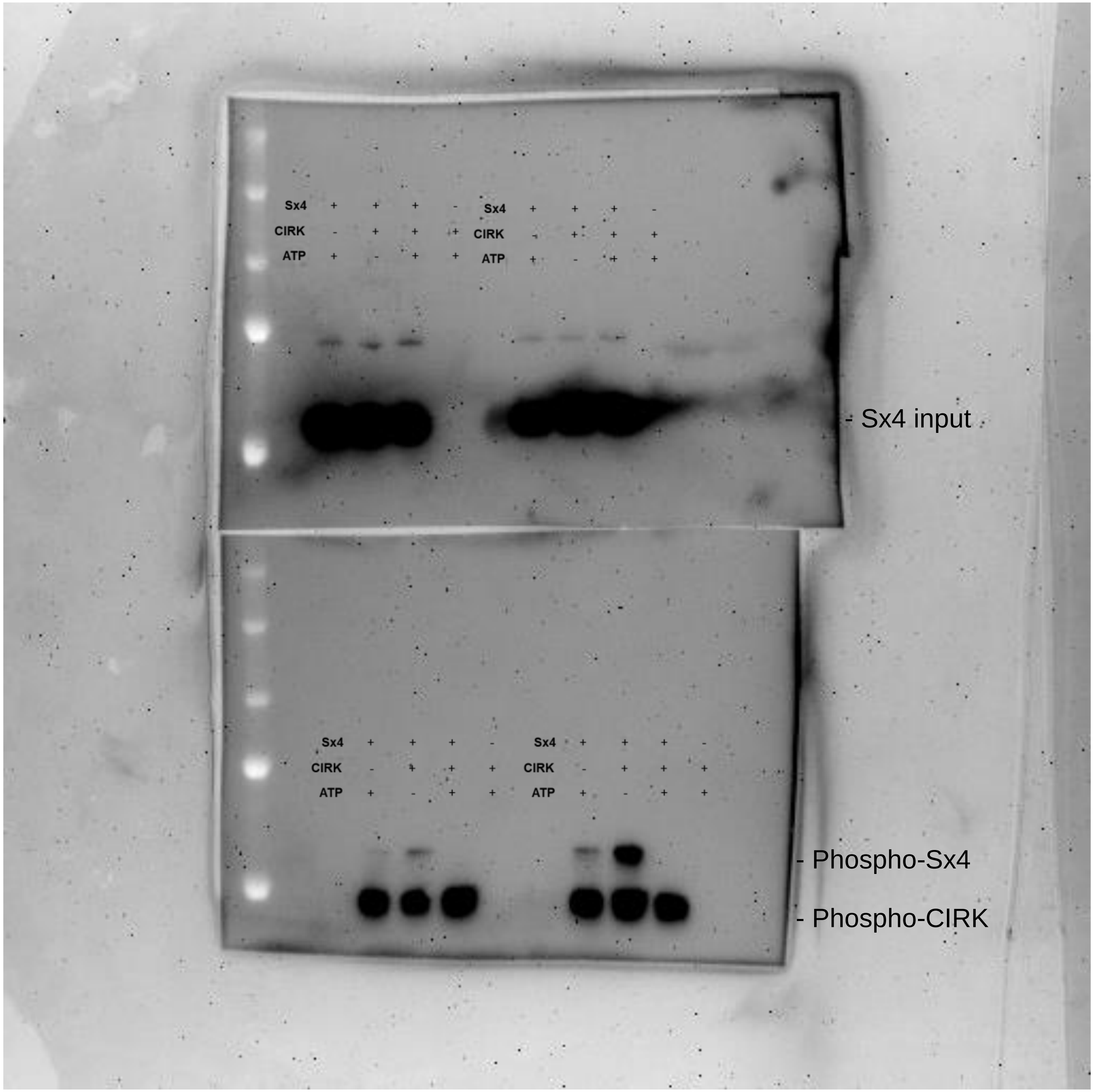

B

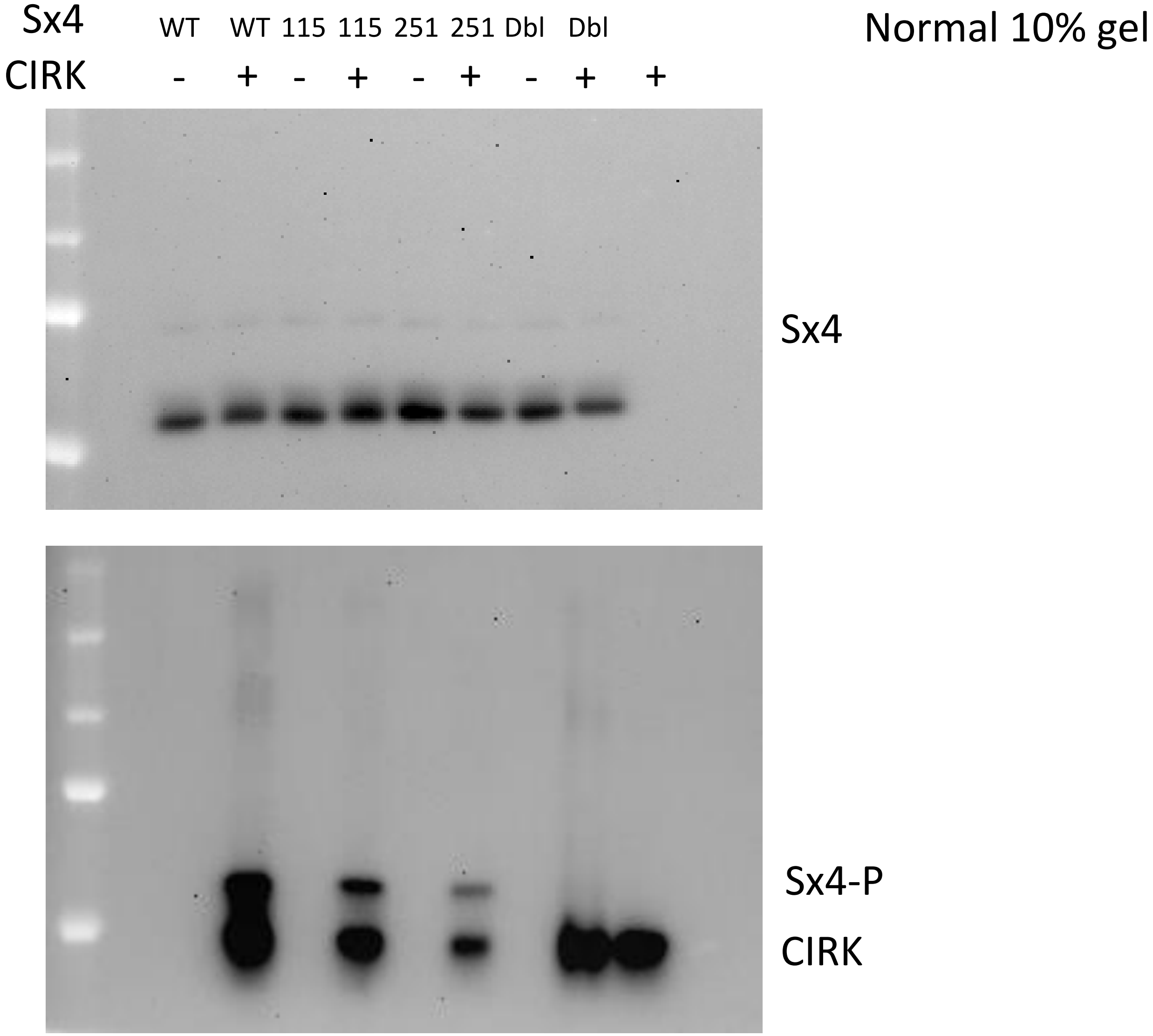
Sx4
WT WT 115 115 251 251 Dbl Dbl
CIRK
- + - + - + - + +
Normal 10% gel
Sx4
Sx4-P
CIRK

Figure S2. Original Western Blotting Figures of Figure 2A

First assay

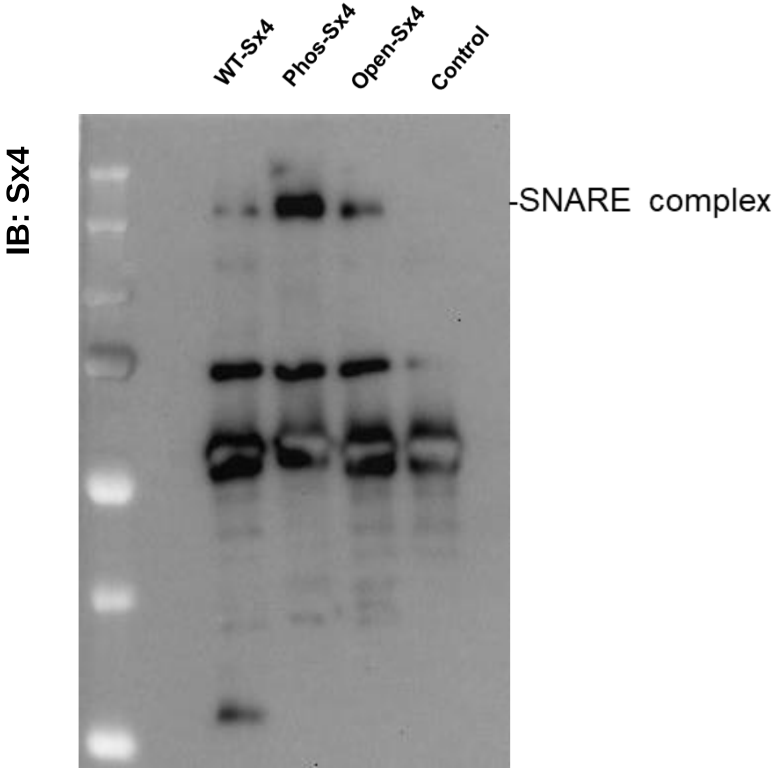

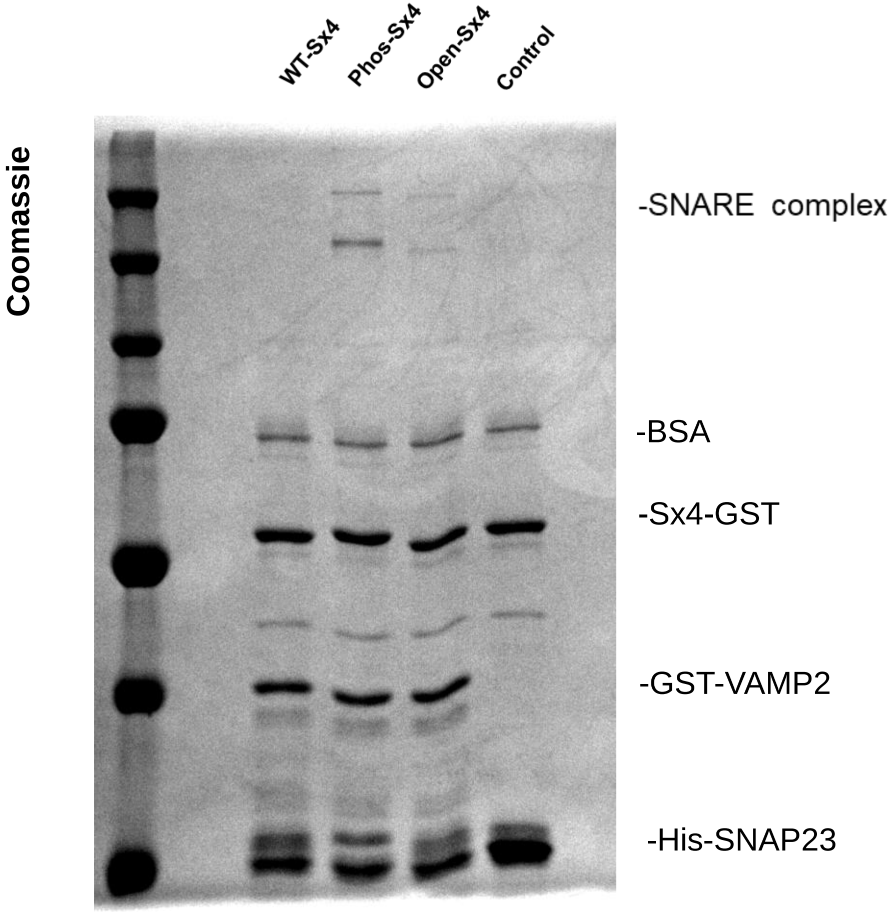

Second assay

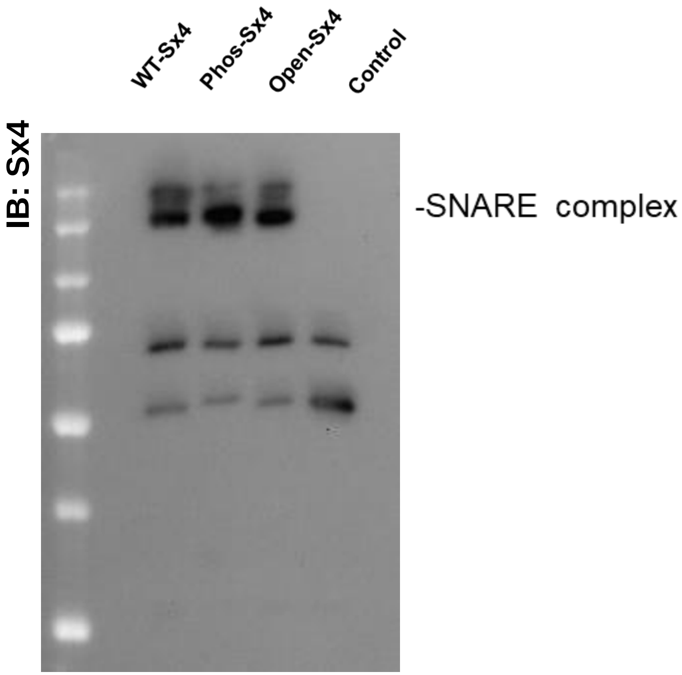

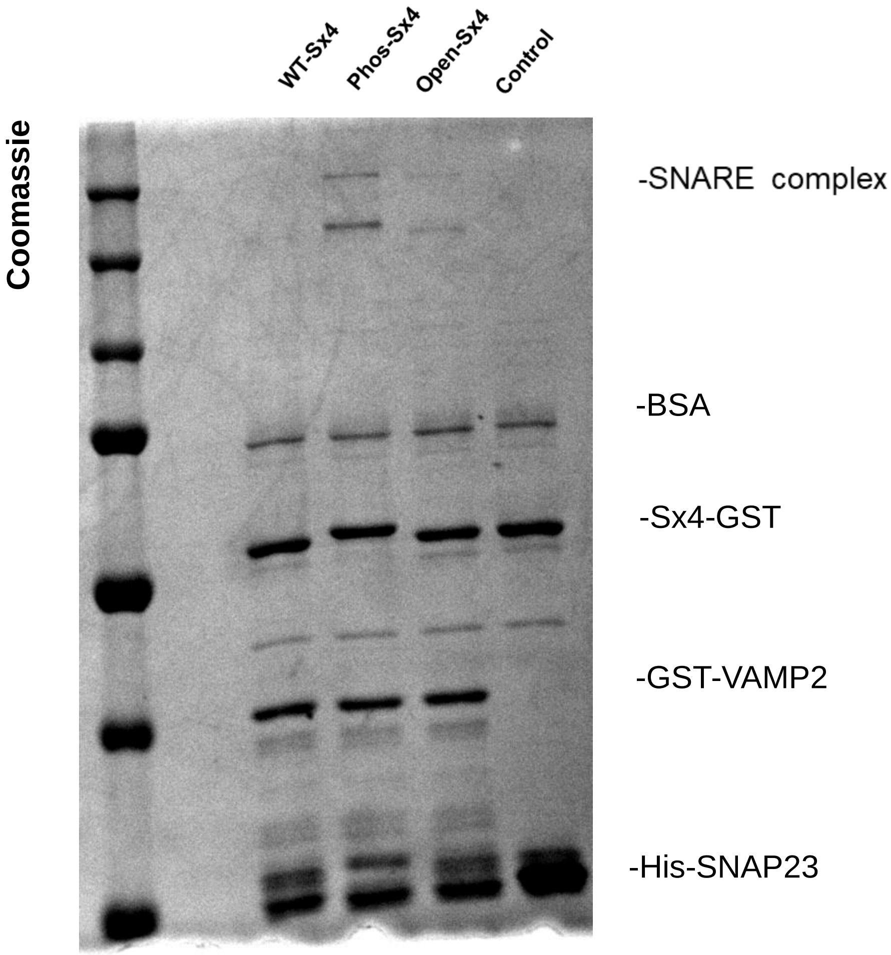

Third assay

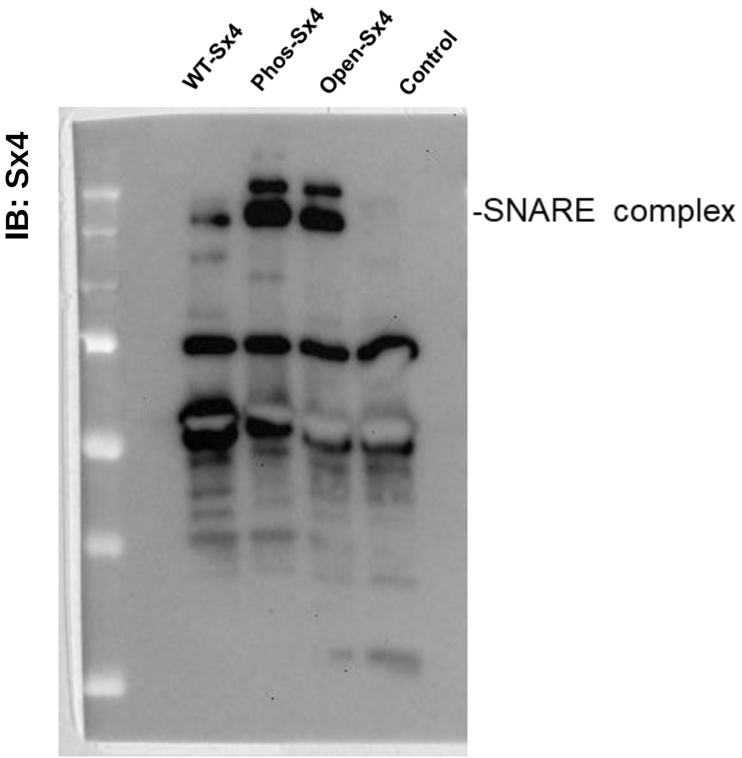
WT-Sx4
Phos-Sx4
Open-Sx4
Control
IB: Sx4
-SNARE complex

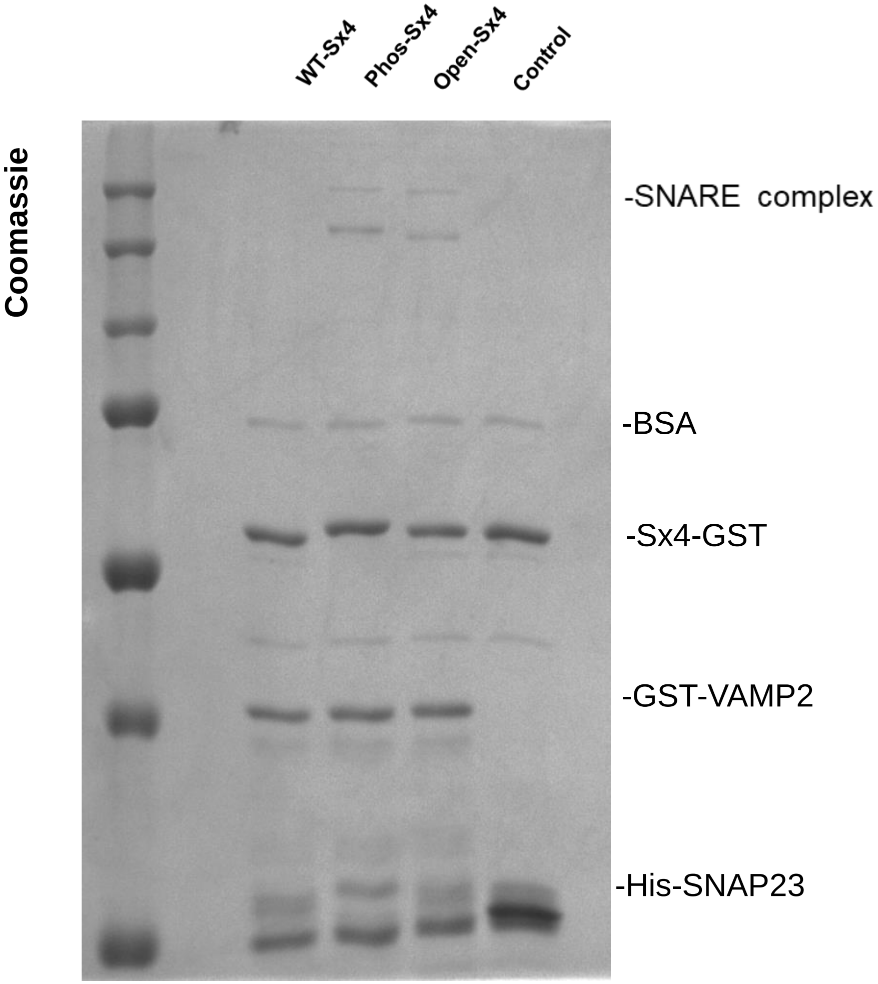
WT-Sx4
Phos-Sx4
Open-Sx4
Control
Coomassie
-SNARE complex
-BSA
-Sx4-GST
-GST-VAMP2
-His-SNAP23

Figure S3. Original Western Blotting Figures of Figure 3A,C,D

First assay

A

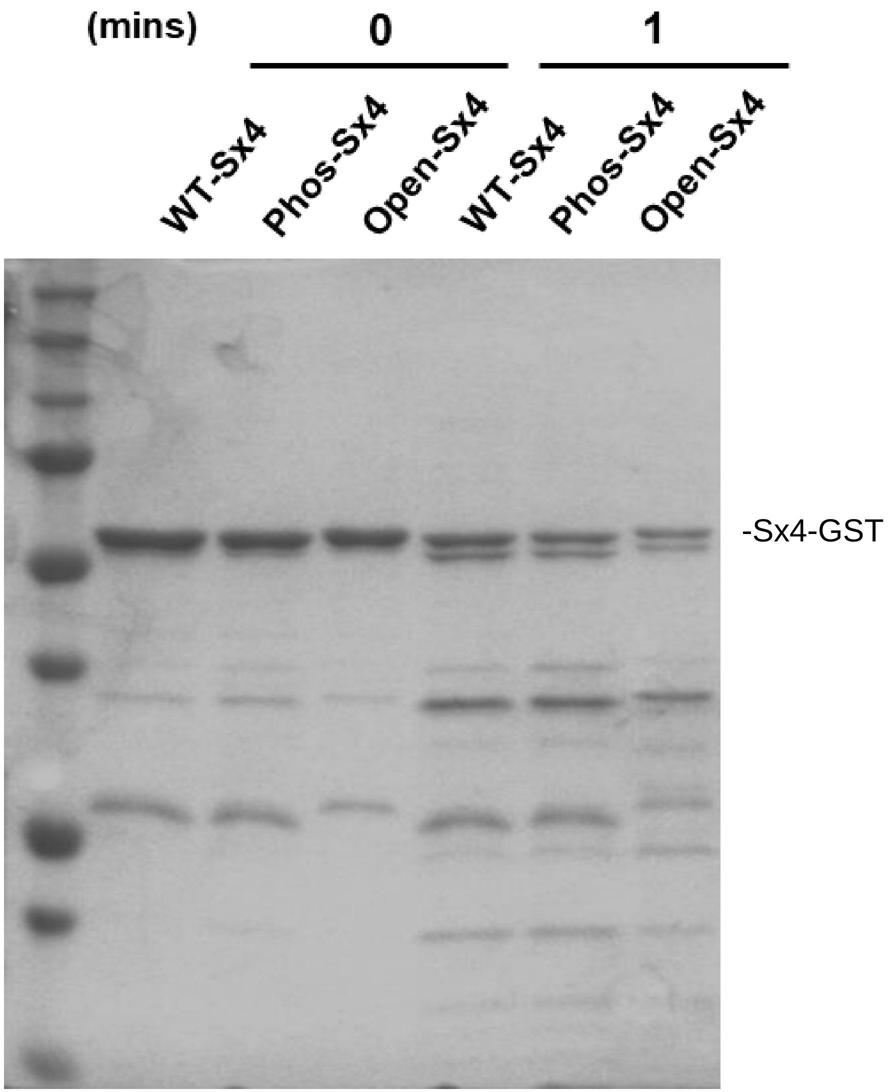

A

Second assay

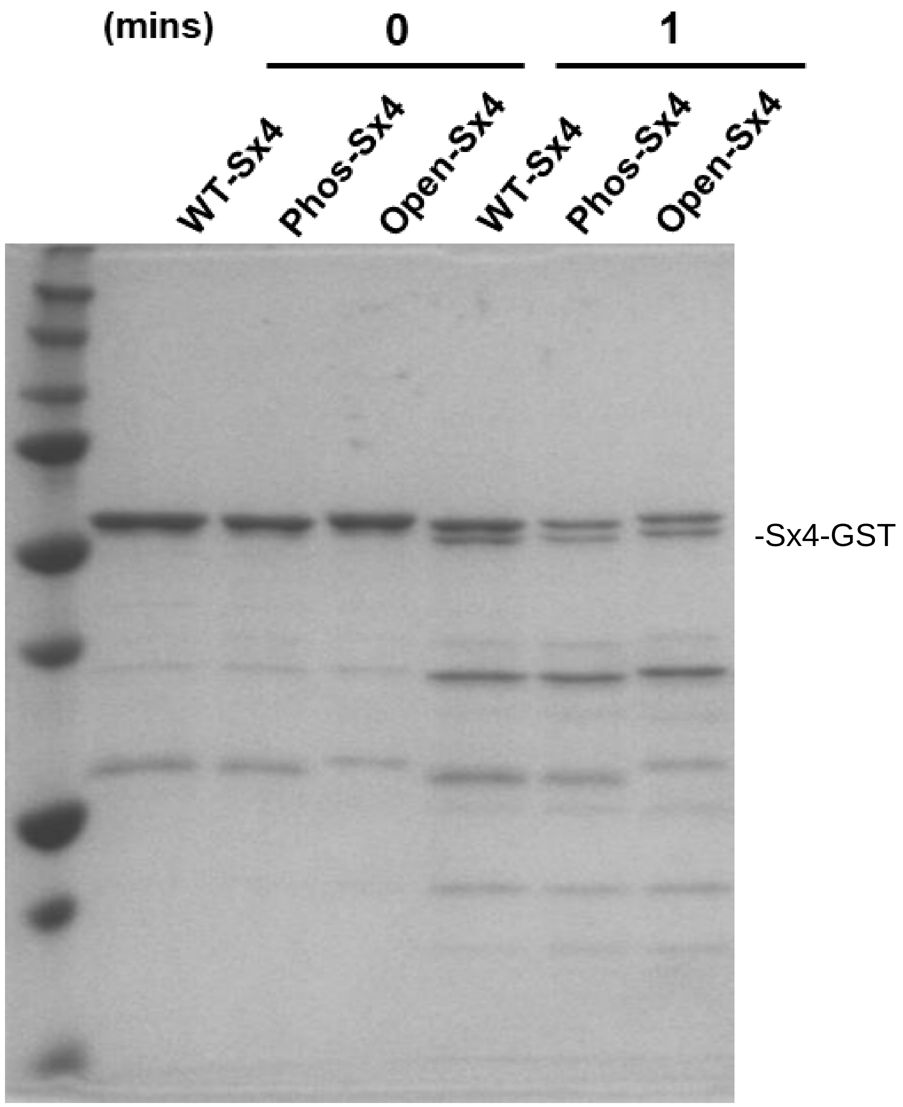

A

Third assay

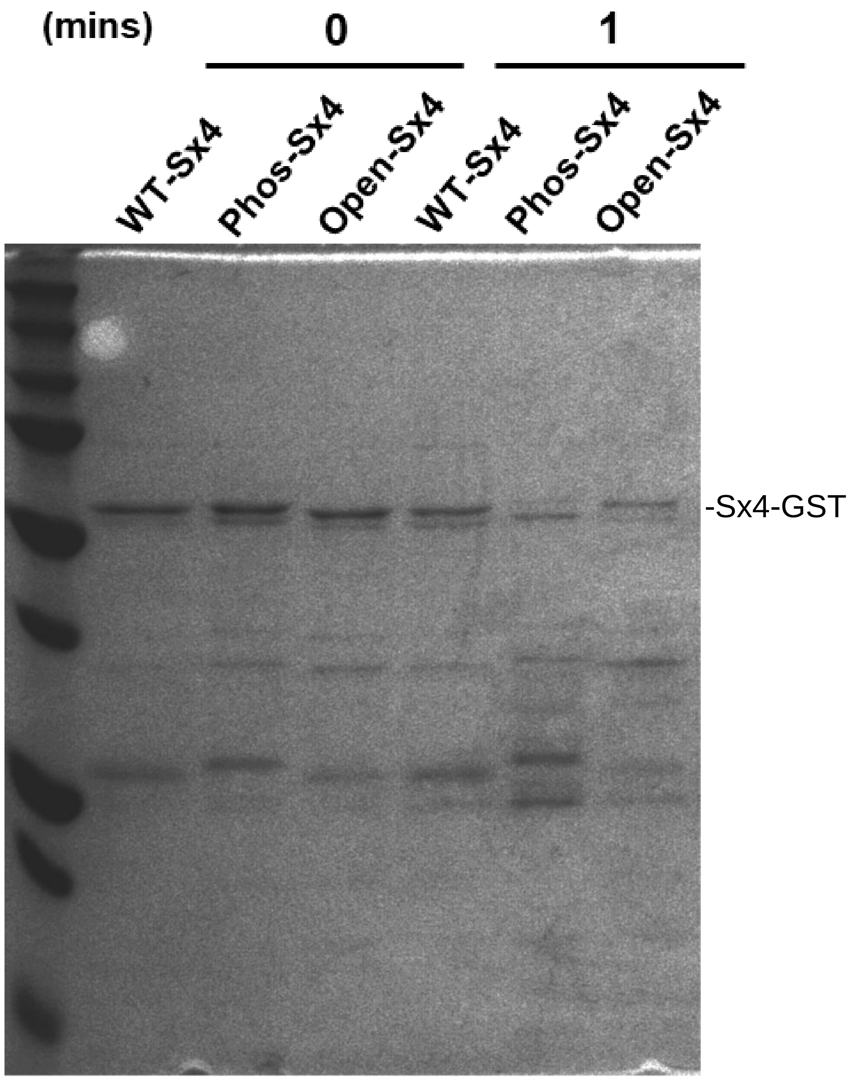

C

First assay

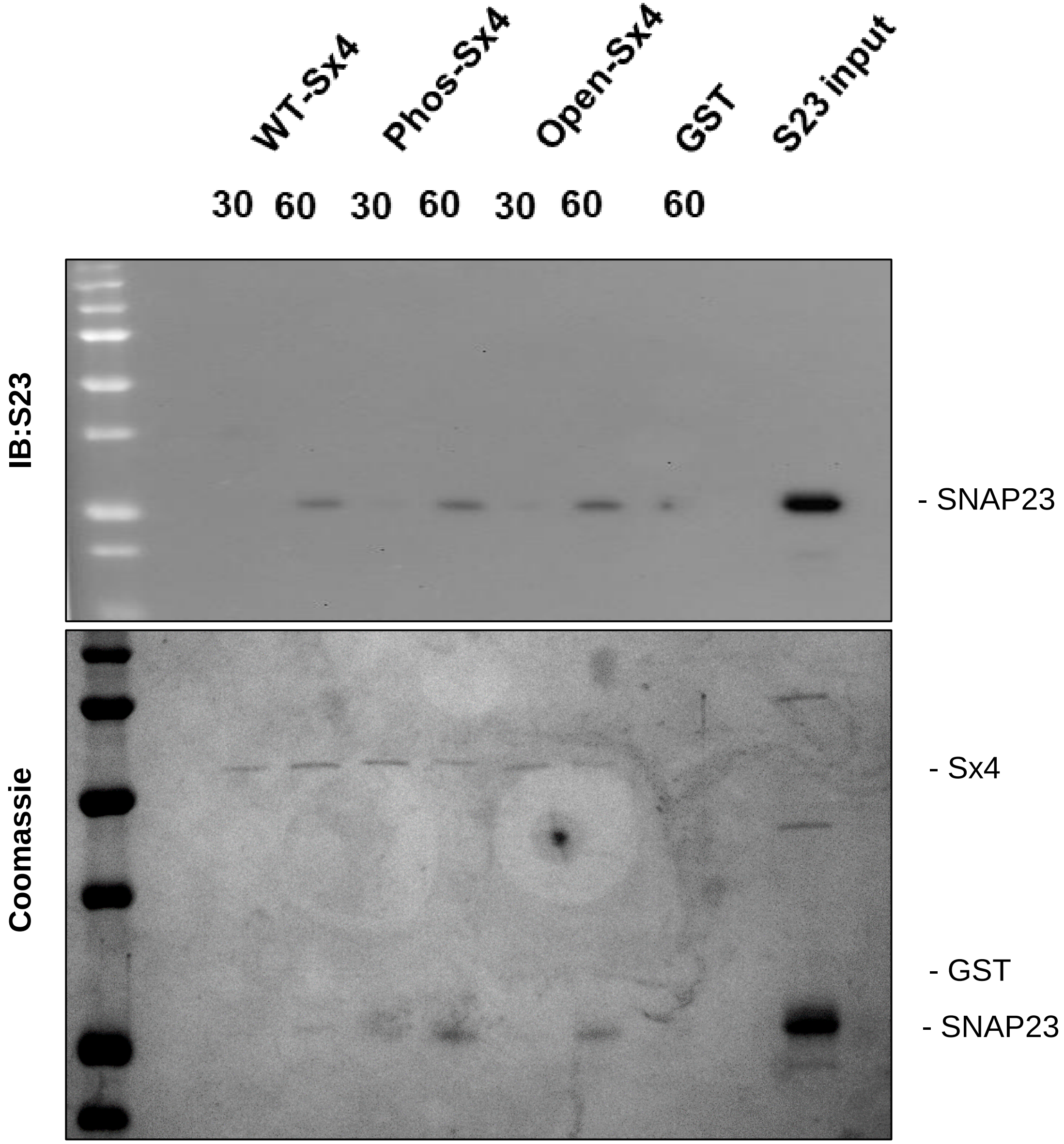

C

Second assay

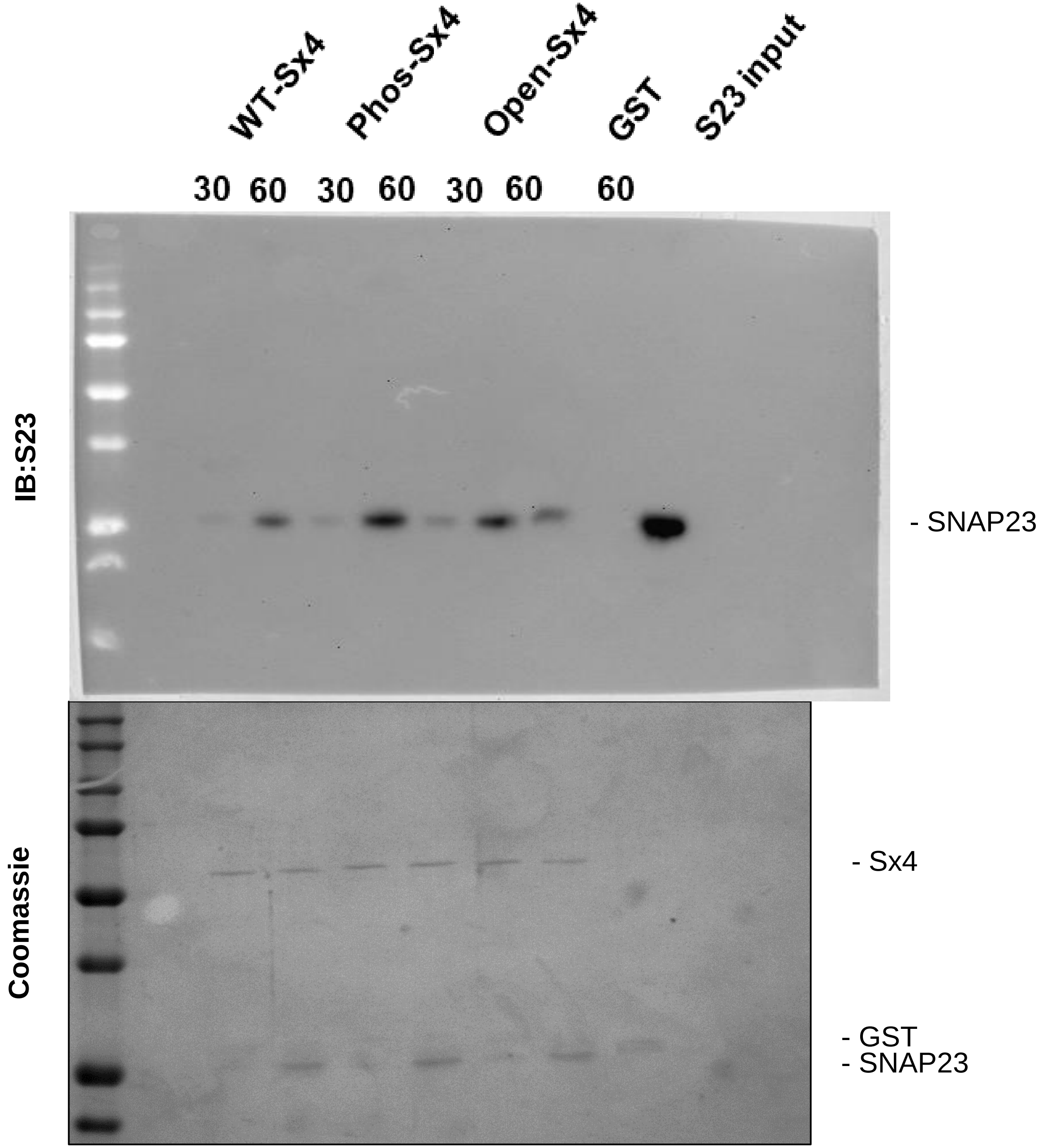

C

Third assay

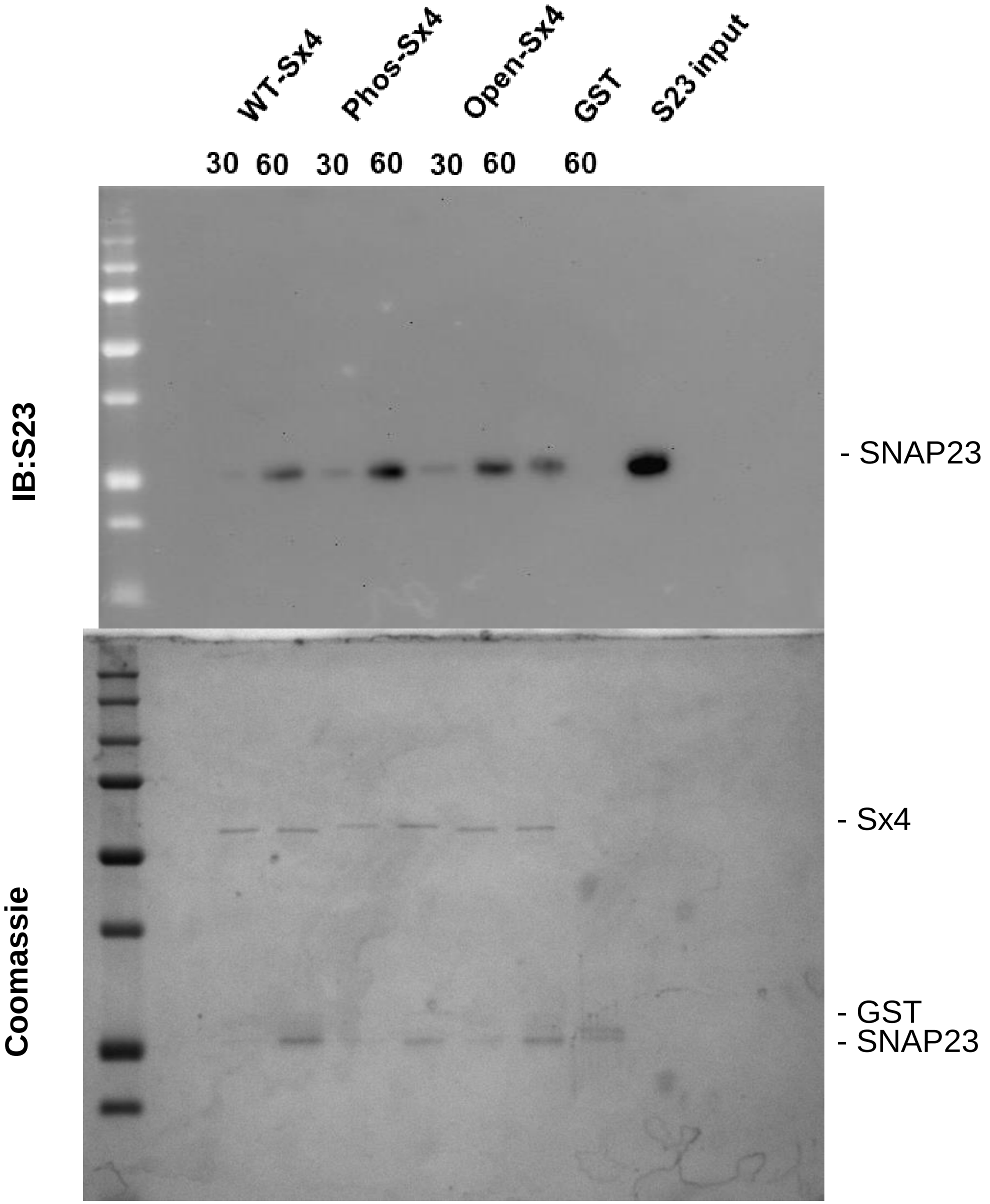

D

First assay

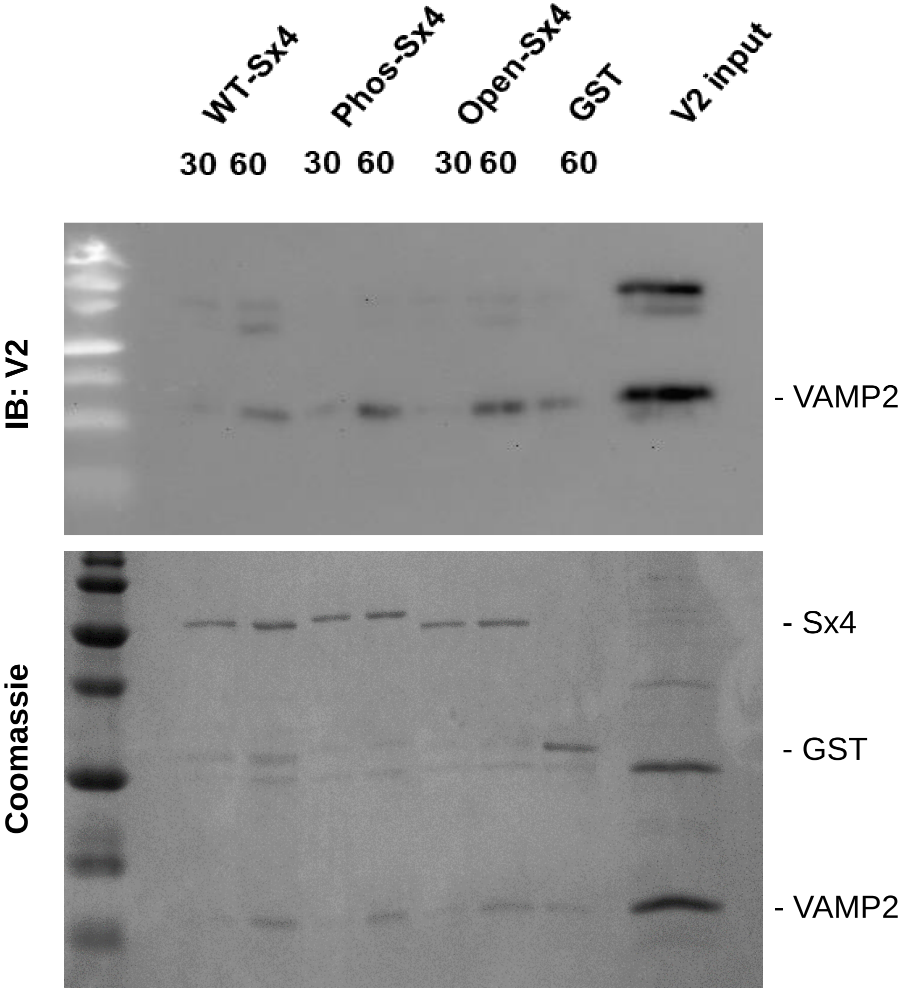

D

Second assay

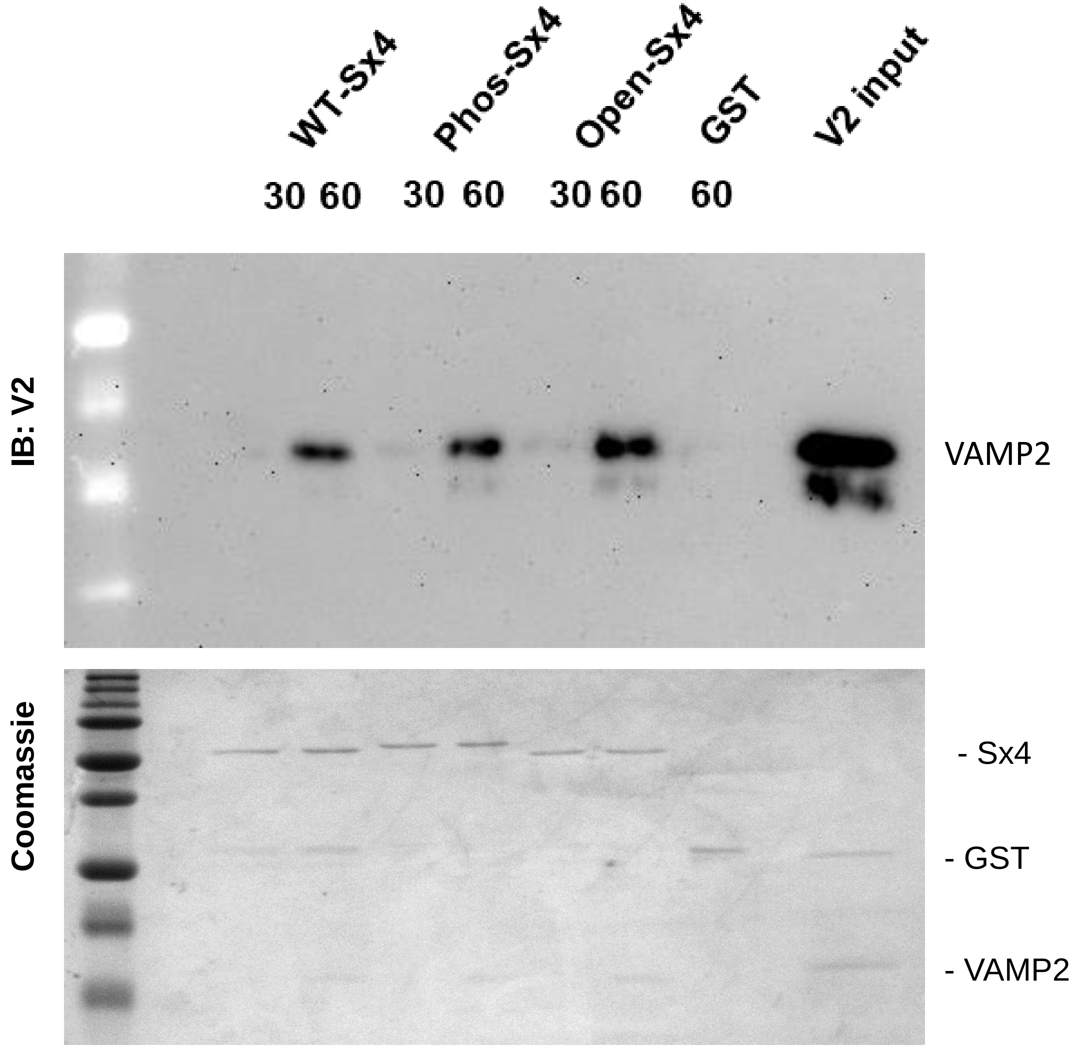

D

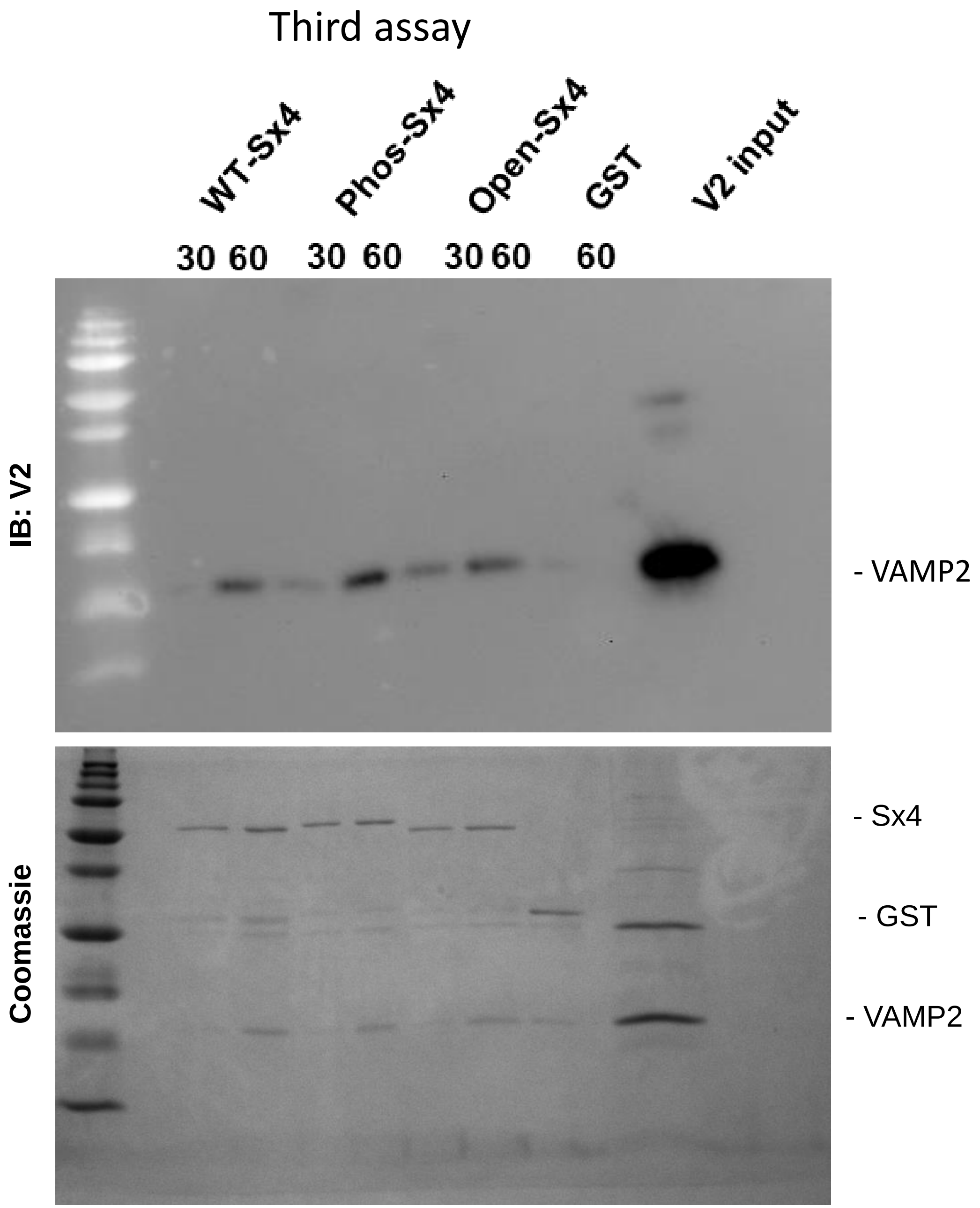
Third assay
WT-Sx4
Phos-Sx4
Open-Sx4
GST
V2 input
30 60
30 60
30 60
60
IB: V2
- VAMP2
Coomassie
- Sx4
- GST
- VAMP2

Figure S4. Original Western Blotting Figures of Figure 5A

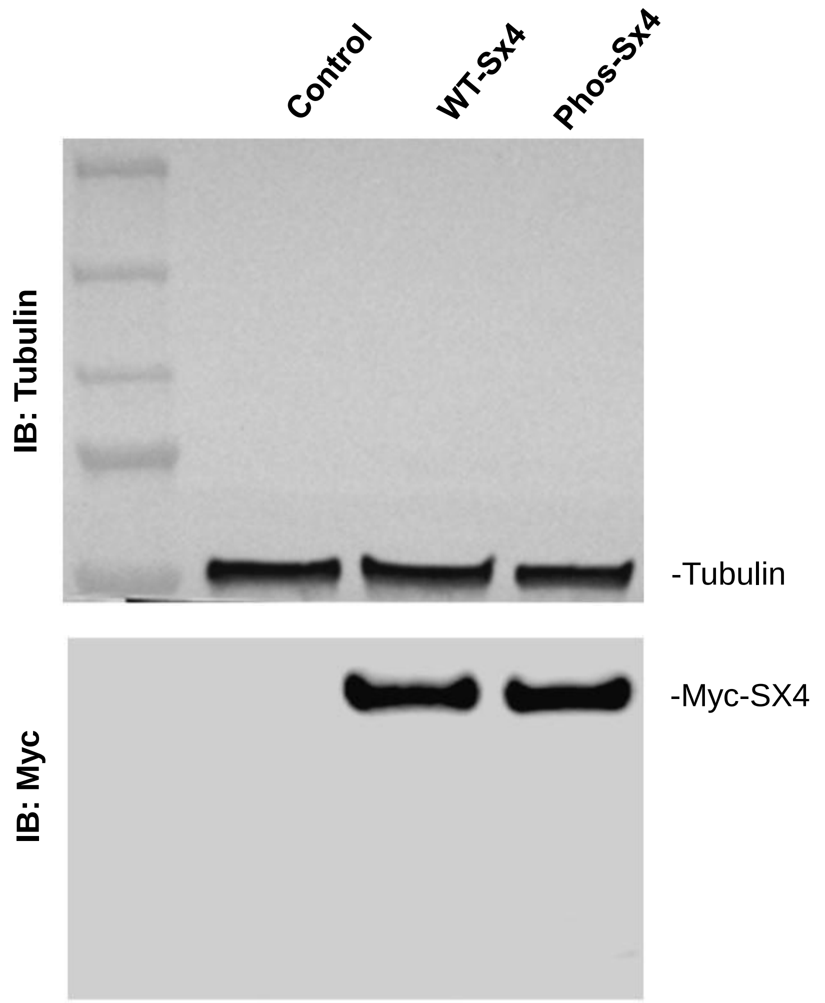

Supplement: Supplementary file 1 [file biomolecules-13-01738-s001.zip › biomolecules-2717853-supplementary.pdf]
